# A Review of Post-Stroke Cognitive Impairment and the Potential Benefits of Stingless Bee Honey Supplementation

**DOI:** 10.21315/mjms2024.31.3.5

**Published:** 2024-06-27

**Authors:** Nor Liyana Ja’afar, Muzaimi Mustapha, Mahaneem Mohamed, Sabarisah Hashim

**Affiliations:** 1 Department of Neurosciences, School of Medical Sciences, Universiti Sains Malaysia, Kota Bharu, Kelantan, Malaysia; 2 Department of Physiology, School of Medical Sciences, Universiti Sains Malaysia, Kota Bharu, Kelantan, Malaysia

**Keywords:** post-stroke cognitive impairment, cognitive function, biomarkers, neuroprotective agent, stingless bee honey

## Abstract

Post-stroke cognitive impairment (PSCI) is a common decline in cognitive abilities that occurs within 3 months after a stroke. During recovery, stroke survivors often experience varying degrees of cognitive decline, with some patients experiencing permanent cognitive deficits. Thus, it is crucial to prioritise recovery and rehabilitation after a stroke to promote optimal protection of and improvement in cognitive function. Honey derived from stingless bees has been linked to various therapeutic properties, including neuroprotective effects. However, scientific evidence for the mechanisms through which these honey supplements enhance cognitive function remains limited. This narrative review aims to provide an overview of the causes of PSCI, current treatments, the biomarkers influencing cognition in post-stroke patients and the potential of stingless bee honey (SBH) as a neuroprotective agent against the progression of PSCI.

## Introduction

Stroke remains the second leading cause of mortality and the third leading cause of disability in the world (1). The World Health Organization (WHO) defines a stroke as ‘rapidly developing clinical signs of focal disturbance of brain function lasting more than 24 h with no apparent cause that is not vascular in origin’ (2). Approximately 80% of strokes are ischaemic; the remainder are haemorrhagic. Meanwhile, transient ischaemic attacks (TIA), often referred as ‘mini strokes’, are a frequently recognised third type of stroke that leave no long-term impact or injury on the brain. Ischaemic stroke can be categorised into four subtypes based on the Trial of Org 10172 in Acute Stroke Treatment (TOAST) classification, including large-artery atherosclerosis, cardioembolism, small-vessel occlusion and stroke of unknown aetiology (3). The clinical signs of stroke, both focal and non-focal, are crucial indicators of the type of stroke, allowing for the timely administration of the appropriate treatment. Stroke generally manifests as an acute focal neurological deficit that might include hemiplegia, speech problems or gait disturbances (4). In Europe, over 1 million stroke patients have been recorded each year, and this number is anticipated to multiply to 1.5 million by 2025 due to the lengthening lifespan of the population (5).

### Post-Stroke Cognitive Impairment

Cognitive decline following a stroke, often referred to as post-stroke cognitive impairment (PSCI), is the typical consequence of stroke. PSCI refers to a spectrum of stroke-related cognitive impairments varying from mild cognitive impairment to dementia (6). The latest meta-analyses report a collective prevalence of PSCI of 53.4%, with mild and major PSCI accounting for 36.4%–38% and 16%, respectively, evaluated within 1.5 years following a stroke (7). Another study found that the frequency of PSCI ranged from 20% to 80%, which differed among nations, races and diagnostic criteria (8). Furthermore, Zhang and Bi (6) mentioned that the incidence of cognitive impairment is more than five to eight times higher than average in stroke patients. PSCI is often underdiagnosed, as it can be overlooked in favour of other health concerns, such as motor or visual symptoms (9). A strong association between PSCI and poor functional outcomes has been documented in one clinical study, although clinical recovery was noticeable in the following weeks and months (10). The progression of cognitive decline is determined by multiple variables, including the period of the stroke, the patient’s age, the location, volume, intensity and extent of neuronal degeneration, the location and size of the post-stroke infarct, the time interval after the stroke, previously established cognitive impairments and other cerebral dysfunctions that predated the event (11).

### Pathophysiology of Post-Stroke Cognitive Impairment

**Figure 1:**
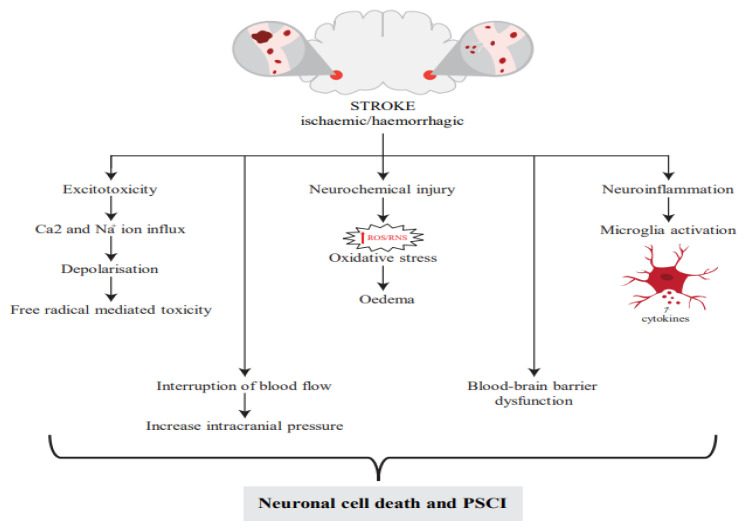
The mechanism of PSCI. Figure adapted from Kuriakose and Xiao (25)

Cerebral injuries such as ischaemic or haemorrhagic events interrupt blood reperfusion, resulting in hypoxia of the brain. As a result, the affected brain region is unable to maintain its reduction-oxidation and ion balance (Figure 1). This disruption affects the electrochemistry of cells, their metabolism and the release of harmful substances. As a result of a significant efflux of potassium ions (K^+^) and an influx of sodium ions (Na^+^), water and calcium ions (Ca^2+^), a process called anoxic depolarisation occurs, along with other related processes (12). These events lead to oxidative and nitrosative stress, excitotoxicity, inflammation and apoptosis, ultimately causing damage to neurons, glial cells and endothelial cells (13–15). Throughout this process, various free radicals are generated, including reactive oxygen species (ROS) and reactive nitrogen species (RNS). These radicals contribute to the breakdown of antioxidant systems and result in brain damage through cerebral ischaemia-reperfusion injury (16). Oxidative stress resulting from the surplus of ROS plays a key role in the pathological process of stroke. The main ROS are superoxide anions (O_2−_), hydroxyl radicals (OH−) and hydrogen peroxide (H_2_O_2_) (16), which stem from the activity of mitochondria, cyclooxygenases, lipoxygenases, nitric oxide synthases (NOSs), NADPH oxidase (NOX) and xanthine oxidase (17).

Overexpression of ROS also leads to neuroinflammation due to microglia (resident macrophage) and astrocyte activation and the attraction of infiltrating leukocytes from the circulating blood (18). These cells increase the number of molecules of the major histocompatibility complex Class II (e.g. B cells, dendritic cells and phagocytes) and cytokines (e.g. interleukins [ILs] and tumour necrosis factor [TNF]) (19). Following microglial activation, the release of pro-inflammatory mediators from the microglia promotes blood-brain barrier (BBB) permeability. In conjunction with chemokine secretion, this process facilitates the sequential infiltration of systemic leukocytes, including neutrophils, macrophages and lymphocytes, all of which exhibit various functional similarities to microglia (20).

In addition, lesion location is a major determinant of PSCI. The precise associations between brain structure and PSCI have likely been seen as weakly correlated because previous studies have employed crude measures. According to previous data, patients with vascular lesions had a slightly higher mortality rate than those without lesions (19% and 14%, respectively) (21). Either ischaemic or haemorrhagic stroke initiated by a vascular lesion and subarachnoid haemorrhage involving the hippocampus and frontotemporal regions has been shown to cause vascular cognitive impairment with deficits in visuospatial memory and language (22). Clinical evidence, such as covert cerebral infarcts, white matter lesions (WMLs), small blood vessel haemorrhages and cortical microinfarcts, frequently contributes to cognitive outcomes among stroke patients (23). WMLs and lacunar stroke instigated by ischaemic damage of small-vessel cerebrovascular disease (24) are cognitive decline predictors and are associated with the severity of cognitive impairment.

### Symptoms and Cognitive Domains Affected

Stroke can be classified based on the lesion’s location and the brain’s arterial vascular territory. There are two major categories of stroke: the first category is anterior (carotid) artery circulation, which includes the middle cerebral artery (MCA) area. About 85% of these strokes are ischaemic and usually result in aphasia, hemiparesis or hemiplegia, loss or disruption of semi-sensory perception, homonymous hemianopia, parietal lobe dysfunction (e.g. astereognosis, agraphaesthesia, impaired two-point discrimination, sensory and visual inattention, left-right dissociation and acalculia) (26). Patients with anterior cerebral artery (ACA) stroke are mostly ambulant, but lower limb weakness is common among those affected (27). Secondly, the stroke occurs in the circulation of the posterior (or vertebrobasilar) artery, which is responsible for 20% of all strokes and supplies the posterior region of the brain, including the brainstem, thalamus, cerebellum and areas of the occipital and temporal lobes. Clinically, most afflicted patients may develop homonymous hemianopia, cortical blindness, ataxia, vertigo, dysarthria, diplopia, dysphagia, Horner’s syndrome, hemiparesis or hemisensory loss contralateral to cranial nerve palsy and cerebellar signs (28). The domains implicated in the progression of PSCI can differ based on various parameters, such as the type, volume, number, location and severity of stroke (29).

Stephens et al. (30) suggested that attention and executive functions are often impaired in post-stroke patients, while failure in memory, orientation and language are stronger indicators of cognitive impairment. The study also highlighted that although early manifestations of attentional and executive impairments were observed, the deficits in memory and language were more suggestive of progressive cognitive decline and dementia. Consistent with other research, attention and executive function impairments were frequently detected, were the most severe following stroke and persisted for an extended period (31).

The affected sites comprise a dominant hemisphere and lesions that impact the prefrontal–subcortical network, inducing failure in executive functions (32). Frontal lobe-related capabilities linked to processing speed, reaction time, working memory and executive activities are most significantly impaired (30). A single major cortico-subcortical ischaemic brain injury can cause acute cognitive decline if it occurs in a region that is functionally vital for cognitive function (33). Meanwhile, major cognitive impairment (dementia) can be caused by an injury to the Papez circuit, which is crucial for memory and emotional control or the Yokovlev circuit (29).

Additionally, a study on PSCI that employed a standardised approach and individual participant data (IPD) from international cohorts in the Stroke and Cognition Consortium (STROKOG) identified a high prevalence of impairments in global cognition and in the five frequently tested domains of attention, memory, language, perception and motor, and executive functions (34). Further, a prior clinical study revealed that executive functions and language improved over time in all stroke patients measured 3 months–18 months post-stroke (35). The study also stated that patients with small vessel disorder had better attentional outcomes than those with cortical strokes. Meanwhile, Gottesman and Hillis (36) reported that aphasia (language impairment) and hemispheric neglect (inability to respond to stimuli on the side opposite the stroke) are the most prevalent cognitive abnormalities following a stroke. According to one study, speed and attention were the most impaired domains. The study reported that visual and verbal memory impairment exhibited no significant difference over time, despite a marked decrease in impairment, from 72.4% in the acute phase to 37.9% at 3 months. Moreover, cognitive deficits such as aphasia and hemispheric neglect were found to be most prevalent amongst stroke patients (31).

### Post-stroke Cognitive Impairment and Current Management

In specialised stroke units, neuroimaging serves as the gold standard for diagnosis, optimal care and the administration of thrombolytic or endovascular treatment. Computed tomography (CT) and magnetic resonance imaging (MRI) techniques, including functional MRI (fMRI) and diffusion tensor imaging (DTI), have been used to screen for lesion areas associated with PSCI (37, 38). These features are crucial in immediate stroke screening and management. The ideal imaging technique for assessing patients with acute stroke should reliably detect both intracranial bleeding and cerebral ischaemia, as well as distinguish cerebrovascular origin from other causes. The CT procedure enables a thorough non-invasive examination that allows for the precise location of vascular blockage and associated haemodynamic tissue conditions in acute stroke patients. However, one study suggested that CT is not sensitive enough for the detection of acute ischaemia and may be inferior to MRI for detecting acute cerebral haemorrhage, owing to extensive inter-rater variation in interpretation (39).

Acute stroke management focuses on stabilising the patient and promptly restoring cerebral perfusion to prevent extensive brain damage. Optimal care necessitates prompt evaluation and intervention to provide maximum reperfusion of brain tissue. The best preventive measures for stroke are administering antiplatelet therapy, treating cardiovascular risk factors (atrial fibrillation and atherosclerosis), optimising treatment for hypertension, dyslipidaemia and diabetes mellitus, as well a smoking cessation (40).

To date, acute treatment of stroke patients has largely followed a wait-and-see approach consisting mainly of supportive care followed by rehabilitation and secondary preventive measures. Over the past two decades, several interventions have been shown to reduce stroke mortality and morbidity. Procedures such as endovascular thrombectomy (EVT) offer selected patients the possibility of a full recovery from an otherwise catastrophic life event. Patients with significant impairments who score from 8–20 on the National Institutes of Health Stroke Scale (NIHSS) are more likely to benefit from reperfusion with EVT (41) The initial few hours following the beginning of symptoms are crucial for achieving optimal stroke treatment. The thrombectomy has a bigger impact in the first 3 h–4.5 h following the stroke compared with delayed recanalisation 5 h–8 h later (42). Prompt response is required to save the at-risk penumbra, which consequently lowers associated morbidity, mortality and long-term disability. Nevertheless, just a tiny fraction of stroke patients are qualified for thrombolysis and recanalisation therapies; thus, stroke prevention continues to be the focus of stroke management (43). Further, cognitive impairment following a stroke is likely to hinder a patient’s adherence to a drug treatment regime, hence complicating optimal stroke therapy.

Thrombolytic therapy (also termed fibrinolytic therapy) involves a class of drugs used to dissolve intravascular clots or inhibit the formation of new blood clots (44). Currently, recombinant tissue plasminogen activator (r-tPA) is the sole therapy that has been approved by the U.S. Food and Drug Administration (FDA) for ischaemic stroke (45). Antiplatelet drugs, including aspirin, can reduce the occurrence of ischaemic stroke, preferably in large artery atherosclerosis patients (46). On the other hand, there is no effective treatment for haemorrhagic stroke (47) Therefore, the rapid and accurate identification of the type of stroke upon admission increases the proportion of ischemic stroke patients receiving r-tPA therapy, consequently improving clinical outcomes for many stroke patients. The preliminary evaluation of a patient who is suspected to have had a stroke should be conducted instantly to guarantee maximum restoration of blood flow to the cerebral tissues. Several steps for the first screening are i) the exclusion of intracranial haemorrhage, ii) checking for thrombolytic contraindications and iii) the characterisation of the infarct. History taking (especially the time of onset of neurological symptoms) should first be conducted by the doctors, followed by physical examination and neuroimaging analysis (to exclude haemorrhagic factors) (40).

Neuropsychological tests, for example, the Mini-Mental State Examination (MMSE), Information Questionnaire on Cognitive Decline in the Elderly (IQCODE) (48), the National Institute of Neurological Disease and Stroke—Canadian Stroke Network (NINDS-CSN) (49), Montreal Cognitive Assessment (MoCA) (50), Alzheimer’s Disease Assessment Scale—Cognitive (ADAS-Cog) and a few others (8) can be employed to evaluate the extent of PSCI. The MMSE is extensively used by frontline physicians to detect cognitive decline after a stroke. However, the MMSE has been criticised for failing to adequately account for several components of cognition, and it is unable to consistently identify mild cognitive abnormalities (50). According to a prior study, using the MoCA, which is much more sensitive to mild cognitive impairment than the MMSE, is one way of circumventing the limitations of the latter (51).

### Risk Factors for Post-Stroke Cognitive Impairment

The current understanding of the underlying mechanisms of PSCI remains partial at best. The risk of PSCI is related to several demographic variables, as well as vascular factors. Age has the biggest influence on stroke and PSCI. Evidence suggests that after age 65, the prevalence of PSCI rises exponentially with age (52). A recent meta-analysis concluded that age, educational level, hypertension, diabetes, atrial fibrillation, history of stroke, Fazakas score, NIHSS score, hyperhomocysteinaemia and alcohol consumption were strongly correlated with cognitive impairment 3 months–6 months after ischaemic stroke (53). According to a clinical investigation, cognitive impairments were significant among elderly patients who had lower levels of education, atrial fibrillation, a history of stroke, left carotid region infarction, numerous lesions, embolism and dysphasia, as well as used alcohol daily (54). However, the age-specific incidence of major PSCI, such as dementia, has decreased across countries, likely due to positive progress in health care, education, nutrition and lifestyle choices (55).

Hypertension is a leading preventable determinant of mild and major PSCI. It has been stated that regulating blood pressure by consuming antihypertensive drugs lowers the risk of developing cognitive abnormalities (56). Antihypertensive medication is recommended for patients who are eligible for IV thrombolysis such that their blood pressure is less than 185/110 mmHg before therapy and less than 180/105 mmHg within 24 h post-treatment (57). Nevertheless, uncertainty persists regarding whether the cognitive benefits are brought about by lowering blood pressure alone or by antihypertensive medications’ direct effects on the brain that are unrelated to blood pressure regulation (58).

Diabetes mellitus (DM) is another well-known modifiable determining factor for stroke and its clinical outcomes. A retrospective study in a biracial population (black and white) postulated that a significant fraction of stroke patients have diabetes, especially among individuals 65 years old and older, regardless of race (59). A stroke can occur if cerebral blood vessels are directly affected by DM, which can cause pathologic alterations in blood vessels at various locations (60). Research has identified significant predictors of poor recovery from diabetes, including increased mortality, particularly after an ischaemic stroke (61).

Atrial fibrillation (AF) is another significant underlying mechanism for stroke, multiplying the risk up to five times, particularly in older individuals (62). Additionally, a meta-analysis concluded that AF is significantly correlated with an increased rate of cognitive impairment and dementia in first-ever or recurring stroke patients (63). AF causes 15% of all strokes and results in more severe disability and higher mortality rates compared to strokes unrelated to AF (64). Patients with AF have been discovered to have decreased blood flow in the left atrium, which results in cerebral thrombolysis and embolism. However, studies have challenged this conclusion, citing scant evidence for the sequential occurrence of AF and stroke, further noting that in some cases, AF is observed only after a stroke has occurred (65).

Stroke-related neuroanatomical lesions manifested in critical sites, including the hippocampus and white matter, as well as cerebral microhaemorrhages (CMBs) due to small cerebrovascular diseases, either from stroke alone or mixed Alzheimer’s disease, contribute to the pathogenesis of PSCI (8). Mijajlović and colleagues (66) also asserted that individuals with mild to moderate stroke who already have WMLs are more likely to experience cognitive impairment, regardless of the presence of new ischaemic lesions.

Natural products, such as herbs, plants and certain foods, are increasingly recognised for their potential to improve cognitive function. Among these, honey, a natural sweetener produced by bees, has emerged as a noteworthy contender, exhibiting a wide range of health benefits and possible effects on cognitive health. Preliminary research indicates that honey might positively influence brain function and memory. While additional studies are necessary to fully describe the underlying mode of action, the inclusion of honey in a balanced diet could bring promising cognitive benefits.

### Honey and Cognitive Health: Treatment and Prevention

Stingless bee honey (SBH) possesses exceptional anti-inflammatory and antimicrobial characteristics (67) and has great potential to halt the progression of PSCI. As the name suggests, the stingless bee species have no sting. Although they are part of the family Apidae, which also includes stinging bees, they belong to a distinct subfamily. While stinging bees are members of the subfamily Apis, the stingless bee is a member of the subfamily Melipona. There are 500 recognised species of stingless bees and 64 different genera widely distributed in the Neotropical, Afrotropical and Indo-Australian regions (68). Folk medicine has long held the idea that honey is a dietary supplement that improves cognitive function. Unfortunately, honey’s nootropic and neuropharmacological potential has not been exhaustively investigated.

SBH contains a number of phenolic compounds, including gallic acid, caffeic acid, catechin, apigenin and flavonoids (69, 70). Phenolic compounds have protective effects against multiple neurodegenerative disorders due to their antioxidant qualities and their ability to influence several cellular-level processes at multiple levels, including enzyme inhibition, gene expression modulation and protein phosphorylation (71). However, it is difficult to pinpoint the precise biological process that underlies each polyphenol and to ascertain how polyphenols impact human health. In a previous study on young rats fed with plant polyphenols xanthohumol, quercetin and a phlorotannin extract for 8 weeks, it was discovered that dietary polyphenols inhibited depression and anxiety-like behaviours in rats induced by maternal separation (72).

A few findings have supported the correlation between honey consumption and enhanced cognitive function. Several pre-clinical trials on the therapeutic properties of honey have revealed that honey consumption improves cognitive function. Chepulis et al. (73) reported that the long-term (12 months) dietary intake of honey reduced anxiety and improved spatial memory in young adult rodents in comparison with those fed sucrose or a sugar-free diet. Similarly, another study demonstrated that metabolic syndrome-induced rats supplemented with SBH exhibited significantly lower anxiety levels and higher memory retention (74). The study asserted that the consumption of SBH rich in antioxidant compounds had a positive effect on brain health by lowering the levels of oxidative compounds in the brain and enhancing the cognitive performance of metabolic disease-induced rats. The study concluded that SBH and its phytochemical properties have highly promising potential as a natural preventive remedy against anxiety and memory loss. This demonstrates the potential value of SBH in attenuating neurodegenerative disorders via the antioxidant impact of its polyphenol content.

In particular, honey may be a promising agent to relieve cognitive decline and improve spatial memory function by reducing oxidative stress, inflammation and apoptosis in neurodegenerative illnesses. Zhang et al. (75) conducted a study in animal models with intracerebral haemorrhage treated with quercetin, demonstrating significant reduction in lesion volume, brain water content and levels of inflammatory biomarkers, along with the quantity of apoptotic cells, notably in the 50 mg/kg quercetin group. These findings support quercetin’s ability to treat brain damage by preventing apoptosis and the inflammatory response, helping to restore nerve function.

### Potential Neuroprotective Mechanism of SBH against PSCI

**Figure 2:**
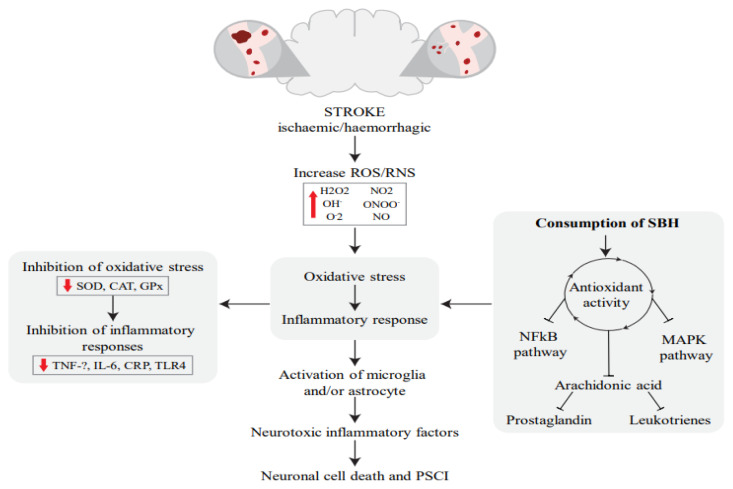
Potential Potential mechanism of action of SBH as an antioxidant to halt the progression of PSCI. Adapted from Hashim et al. (77)

Cerebral artery occlusion leads to oxygen, glucose and lipid deprivation, resulting in necrosis of the cerebral parenchyma. Various mechanisms, such as excitotoxicity, oxidative stress and inflammation, have been proposed to explain brain injury stemming from an ischaemic event (76). In recent years, research has shown that SBH has potent antioxidant and anti-inflammatory effects, making it a valuable natural remedy for cognitive decline resulting from stroke. The potential mechanisms of action of SBH against PSCI are shown in Figure 2. The antioxidant activity of SBH inhibits two important signalling pathways: the nuclear factor kappa-light-chain-enhancer of activated B cells (NFκB) and mitogen-activated protein kinases (MAPK). This suppresses the genes responsible for the production of inflammatory factors, leading to a decrease in the production of pro-inflammatory cytokines and chemokines (e.g. TNF-α, IL-6 and CRP). Inhibition of these signalling pathways also suppresses oxidative stress, which is reflected in the reduced expression of oxidative biomarkers (superoxide dismutase [SOD], catalase [CAT] and glutathione peroxidase [GPx]). In addition, the antioxidant phenolic compounds of SBH can reduce the release of arachidonic acids during the oxidation of membrane phospholipids, thus reducing their metabolites, such as leukotrienes and prostaglandins, which are considered important mediators of inflammation.

Overall, SBH is a potential complementary agent for reducing neuroinflammation and preventing the rapid progression of PSCI due to its biochemical action. Furthermore, exploring the use of SBH to enhance cognitive performance presents an intriguing avenue for future investigation. SBH has demonstrated its ability to regulate biomarkers that play a vital role in cognitive processes. The modulation of these biomarkers by SBH suggests its potential as a natural treatment for PSCI and to improve overall cognitive function. However, extensive research is necessary to precisely elucidate the intricate mechanisms underlying the impact of honey on cognitive biomarkers, thus solidifying its role as a viable therapeutic option.

### Biomarkers Associated with Cognitive Performance

As noted above, various cognitive tests are frequently used to evaluate PSCI (e.g. the MMSE, NINDS-CSN and MoCA). The results of these tests, however, may not be sufficient for the diagnosis and prognosis of PSCI because they are subjective and inconclusive. Moreover, neuroimaging procedures (e.g. MRI and CT) that are frequently employed following brain damage are inadequate to identify the precise systemic physiological mechanisms underpinning the brain’s recovery (78). In recent years, molecular biomarkers for stroke have gradually attracted global interest as they facilitate diagnosis, characterise the severity of clinical outcomes, estimate long-term prognosis and determine appropriate treatment options.

An increasing body of research has reported a correlation between cognitive impairment following stroke and alterations in the production of biomarkers, including C-reactive protein (CRP), interleukin 6 (IL-6) and interleukin 10 (IL-10), which are found in blood, urine and other body fluids (Table 1). Hence, the detection of biomarkers in circulating blood serum, plasma and cerebrospinal fluid (CSF) has the potential to improve the accuracy of diagnosis and prognosis of PSCI (6). In addition, a clinical study showed lower expression of soluble receptors for advanced glycation end products (sRAGE), as well as higher levels of β-secretase enzyme (BACE1) and neprilysin (NEP) in dementia patients (79). However, the regression analysis indicated that sRAGE and BACE1 were the only biomarkers that exhibited changes preceding cognitive impairment after a stroke. In short, only the genotypes of BACE1 and sRAGE could be biomarkers used to diagnose PSCI. A recent meta-analysis by Kim et al. (80) reported significantly high levels of homocysteine (Hcy), CRP, total cholesterol (TC) and low-density lipoprotein cholesterol (LDL-C) detected in PSCI patients in comparison to the non-PSCI group, suggesting that these blood proteins hold promise as potential biomarkers for PSCI.

The advancement of neurodegenerative diseases is closely related to a surplus of ROS and the production of proinflammatory mediators and cytokines. Glutamate is the most abundant excitatory amino acid and a key neurotransmitter in the central nervous system (CNS) that mediates neuronal response to various situations, including hyperglycaemia, ischaemia and retinal injury. This neurotransmitter contributes to CNS conditions found in Alzheimer’s disease, Parkinson’s disease, glaucoma and diabetic retinopathy (81). Moreover, several neuropsychiatric illnesses, including schizophrenia and depression, have been linked to abnormal glutamate activity, which was poorly understood until recently (82). This warrants further exploration of the relationship between the pathogenesis and pathophysiology of numerous neuropsychiatric disorders and the action of glutamate.

In addition, a recent Norwegian Cognitive Impairment after Stroke study reported that higher concentrations of inflammatory biomarkers in plasma were associated with lower MoCA scores up to 36 months after a stroke. This association was most pronounced for inflammatory biomarkers measured in the acute phase after a stroke (83). This further confirmed the multifaceted clinical utility of biomarkers for the detection and treatment of PSCI. Early detection and prediction of PSCI can enable timely interventions and support cognitive rehabilitation strategies. Biomarkers can aid in stratifying PSCI subtypes and assessing the severity of cognitive impairment, allowing for tailored treatment approaches. They also have potential applications in monitoring treatment response and disease progression, providing valuable information for clinical decision-making. Furthermore, biomarkers may guide researchers in identifying potential therapeutic targets for intervention and developing novel treatments specifically targeting the underlying pathophysiological processes.

## Conclusion

Stroke and PSCI prevention continues to be a key component of any plan for achieving ideal brain health. In order to formulate effective preventive and treatment measures, it will be crucial to comprehend the role of preventable risk factors in the emergence of cognitive impairment due to stroke. Further studies are proposed to investigate the relationship between oxidative (SOD, CAT and GPx) and inflammatory biomarkers (especially IL-6, TNFα, TLR4, CRP, etc.) and the use of SBH as an additional treatment modality in the early stages of PSCI.

## Figures and Tables

**Table 1:** Biomarkers associated with post-stroke cognitive impairment

References	Biomarker(s)	Population	Results
84	CRP	30 stroke patients and 30 healthy subjects matched for age, sex and level of education	In comparison to healthy subjects, stroke survivors displayed markedly elevated levels of serum hsCRP
85	IL-6, IL-10, IL-1β and TNF-α	92 patients with AIS and 14 healthy subjects of the same age	All patients had a higher serum level IL-10 than the control group. Patients with dysexecutive cognitive impairment (DCI) had higher levels of IL-1β and IL-10 in CSF and IL-6 in serum than patients with normal cognition (NC). A non-significant increase in serum TNFα levels was observed in DCI patients compared to NC stroke patients
86	CRP, IL-1β, IL-6, IL-8, IL-10, IL-12 and TNF-α	243 ischaemic stroke patients	At baseline, elevated serum IL-8 levels were independently linked to cognitive impairment, whereas higher serum IL-12 levels were associated with subsequent cognitive decline
87	Malondialdehyde (MDA) and 8 hydroxydeoxyquanosine (8-OHdG)	101 patients with PSCI, 92 non-PSCI patients	Serum levels of 8-OHdG and MDA were both significantly higher in the PSCI group than in the non-PSCI group
88	Serum uric acid (UA)	274 patients with acute cerebral infarction (188 exhibited PSCI and 86 exhibited normal cognition)	Serum UA levels were significantly higher in the PSCI group than in the non-PSCI group
89	MicroRNA (miR)-135b-5p and mineralocorticoid receptor (NR3C2)	12 male Sprague Dawley rats modelled via MCA occlusion and 12 rats without MCA occlusion	Overexpression of miR-135b-5p in serum may reduce neuronal damage and inflammatory response in PSCI by targeting NR3C2, which may be useful for the treatment of PSCI
90	Homocysteine	1,070 participants with acute minor ischaemic stroke or transient ischaemic attack and baseline homocysteine information from a nationwide multicentre prospective registry study in China	Women had lower baseline homocysteine levels than men. Elevated baseline homocysteine levels significantly increased the 12-month PSCI risk in women but not in men (odds ratio [OR] 0.86; 95% CI: 0.49, 1.49]; *P* = 0.586)
91	Plasma Aβ-40, Aβ-42, total tau, phosphorylated tau 181 (p-tau181) and BDNF	136 patients with AIS	The p-tau181 level demonstrated potential for enhanced discrimination of PSCI at 3 and 12 months. Among the subjects, the plasma p-tau181 level was highest in those without PSCI, followed by individuals with delayed-onset PSCI and early-onset PSCI with regression. Conversely, subjects with persistent PSCI displayed the lowest significant plasma p-tau181 levels (*P* = 0.0081)
92	Gut microbiota	12 patients with PSCI, 12 patients without PSCI and 12 healthy volunteers	The PSCI group exhibited a notably elevated relative abundance of actinomycetes (LDA score > 2). Among the top 10 bacteria at the phylum level, Firmicutes demonstrated the highest diversity across the three groups. The non-PSCI group displayed an increasing trend in the relative abundance of Verrucomicrophyla, while the PSCI group showed an increasing trend in the relative abundance of Actinobacteria
93	Gut microbiota	19 healthy controls, 27 stroke patients, 29 PSCI patients and 20 post-ST affective disorder patients	Compared to non-PSCI patients, those with PSCI exhibited a significantly higher concentration of Enterococcus, Bacteroides and Escherichia-Shigella, while displaying a lower proportion of Faecalibacterium
94	Serum SOD, erythrocyte sedimentation rate (ESR), CRP and IL-6	187 patients diagnosed with mild AIS	Low serum level SOD was associated with higher inflammatory biomarkers (ESR, CRP and IL-6). Low serum level SOD at baseline was independently associated with a high risk of cognitive impairment at week 2 and after 3 months. Linear associations were observed between serum SOD and cognitive impairment at baseline and 3 months. SOD was identified as a protective factor for cognitive recovery after stroke (OR 1.04; 95% CI: 1.01, 1.08; *P* = 0.024)
95	Serum miR-21, miR-124, miR-132 and miR-200b	45 PSCI and 32 post-stroke cognitive normality (PSCN) patients	PSCI patients displayed higher expression levels of miR-21, miR-132 and miR-200b compared to PSCN patients. Notably, the miR-21 level exhibited a significant positive correlation with the MMSE scores (*r* = 0.752, *P* < 0.001)
96	miR-132	39 subjects with PSCI, 37 subjects with post-stroke cognitive normality (PSCN), 38 age-matched controls (AMC)	The serum miR-132 level of PSCI patients was significantly increased compared to that of PSCN and AMC patients. The miR-132 level correlated with the MoCA score in PSCI patients
97	Serum neuroglobin	316 patients with ICH	Neuroglobin levels in PSCI were significantly different from those in the non-PSCI group. Serum neuroglobin was found to correlate positively with MoCA scores
98	Plasma TC, LDL-C, HDL-C, TG, FBG, HbA1c, serum IGF-1 and VEGF	60 PSCI-patients (30 received recombinant human growth hormone [rhGH] subcutaneously, 30 in placebo group)	Treatment with rhGH improved the lipid profiles (TC, LDL-C, HDL-C and TG), significantly increased IGF 1, but did not significantly change the concentrations of FBG and HbA1c
99	Tissue inhibitor of metalloproteinase-1 (TIMP 1)	598 ischaemic stroke patients	Linear associations were found between TIMP-1 concentrations and cognitive impairment (*P* < 0.01)
100	Galectin-3	416 patients with their first acute ischaemic stroke (PSCI group: 252; non-PSCI group:164)	The PSCI cohort exhibited a significantly higher level of serum galectin-3 compared to the non-PSCI cohort (*P* < 0.001).
